# Current and emerging drugs for Parkinson’s disease: mechanisms, clinical evidence, and future directions

**DOI:** 10.3389/fphar.2026.1805370

**Published:** 2026-06-29

**Authors:** Fang Yang

**Affiliations:** Department of Neurology, Xinchang County People’s Hospital, Shaoxing, Zhejiang, China

**Keywords:** clinical translation, disease-modifying therapy, dopaminergic therapy, neuroprotection, Parkinson’s disease, α-synuclein

## Abstract

Parkinson’s disease (PD) is a progressive neurodegenerative disorder characterized by dopaminergic neuronal loss and α-synuclein pathology, resulting in disabling motor and non-motor symptoms. Pharmacotherapy remains the cornerstone of PD management, yet current treatments are largely symptomatic and fail to halt disease progression. In recent years, substantial advances have been made in both dopaminergic and non-dopaminergic therapeutic strategies. Novel formulations of levodopa and dopamine agonists aim to provide more stable dopaminergic stimulation and reduce motor fluctuations. Meanwhile, emerging non-dopaminergic agents targeting glutamatergic, serotonergic, adenosinergic, and cholinergic systems offer new options for managing dyskinesia, gait impairment, and neuropsychiatric symptoms. Importantly, growing efforts are directed toward disease-modifying therapies, including α-synuclein immunotherapy, inhibitors of protein misfolding and aggregation, glucocerebrosidase-targeted interventions, and neuroprotective agents that modulate mitochondrial dysfunction, oxidative stress, inflammation, and metabolic signaling. Although many candidates have shown encouraging preclinical or early clinical results, definitive disease-modifying efficacy remains to be established. This review summarizes recent progress in PD pharmacotherapy, highlights translational challenges, and discusses future directions toward precision medicine and combination strategies for achieving sustained symptomatic control and disease modification.

## 1 Introduction

Parkinson’s disease (PD) is a progressive neurodegenerative disorder characterized by the selective loss of nigrostriatal dopaminergic neurons and the pathological accumulation of α-synuclein (Di Luca et al., 2022). With an aging global population, the escalating prevalence of PD imposes a substantial clinical and socioeconomic burden, primarily driven by its debilitating motor and non-motor manifestations. Current pharmacological management remains predominantly symptomatic, relying on dopamine replacement strategies such as levodopa, dopamine agonists, and enzyme inhibitors (Li et al., 2025). Although these agents effectively alleviate early motor deficits, their long-term utility is frequently compromised by motor complications, inadequate control of axial symptoms, and the fundamental inability to halt neurodegeneration. Novel levodopa delivery systems, targeted non-dopaminergic modulators, and emerging disease-modifying candidates—including α-synuclein immunotherapies, lysosomal enhancers, and neuroprotective agents—are actively advancing through clinical evaluation. α-Synuclein aggregation is closely linked to mitochondrial dysfunction and neuroinflammation. Misfolded α-synuclein impairs mitochondrial complex I activity, mitophagy, and mitochondrial dynamics, thereby increasing ROS production and oxidative stress. Mitochondrial damage and lysosomal stress further activate the NLRP3 inflammasome and neuroinflammation. Despite promising mechanistic rationale, definitive clinical translation remains constrained by pathophysiological heterogeneity and suboptimal trial endpoints. This review summarizes current and emerging PD pharmacotherapies, critically appraises their clinical evidence and mechanistic foundations, and outlines key translational challenges and future directions toward precision-driven, disease-modifying treatment strategies.

## 2 Dopaminergic symptomatic treatment drugs for Parkinson’s disease

### 2.1 New formulations of levodopa

Recent advances in levodopa delivery systems aim to mitigate pharmacokinetic limitations and provide more continuous striatal dopaminergic stimulation. For rapid rescue of “off” episodes, carbidopa/levodopa inhalation powder (CVT-301) achieves clinical onset within ∼10 min, with maximal benefit observed at 30 min (LeWitt et al., 2019a). To address levodopa’s short half-life and erratic gastrointestinal absorption, several oral modified-release formulations have been developed. Carbidopa/levodopa extended-release capsules (IPX066) integrate immediate- and extended-release granules to stabilize plasma concentrations, significantly reducing daily “off” time compared with conventional immediate-release preparations (Senek et al., 2017). Similarly, the “accordion pill” (AP-CD/LD) utilizes a gastro-retentive polymer film that unfolds in the stomach, enabling sustained drug release over ∼12 h (Murakami et al., 2023; Poewe et al., 2023). Phase II trials demonstrated increased “on” time and reduced “off” time without exacerbating dyskinesia (LeWitt et al., 2019b), with clinical studies ongoing. Additional extended-release candidates, including IPX203 and DM-1992, employ multi-layered tablet or capsule technologies to optimize absorption kinetics and prolong therapeutic exposure (LeWitt et al., 2023; Hauser et al., 2023). For advanced disease with severe motor fluctuations, continuous delivery routes are increasingly utilized. While levodopa/carbidopa intestinal gel is established for duodenal infusion, a triple-combination gel incorporating entacapone is under investigation to further smooth pharmacokinetic profiles and minimize “off” periods. Subcutaneous levodopa infusion (ND0612) maintains stable systemic drug levels, with clinical data confirming pharmacokinetic consistency; injection-site reactions remain the primary adverse effect, and pivotal Phase III trials evaluating clinical efficacy are ongoing (Giladi et al., 2021). Collectively, these innovations seek to bridge the gap between pulsatile oral dosing and continuous dopaminergic stimulation, thereby improving motor control and reducing complication risk.

### 2.2 Novel dopamine receptor agonists/antagonists and new formulations

Dopaminergic therapy emphasizes receptor subtype selectivity and optimized pharmacokinetic profiles. Tavapadon, a selective D1/D5 receptor agonist, demonstrated significant motor improvement in early-stage PD during Phase II trials (Riesenberg et al., 2020). Ongoing clinical studies are evaluating its efficacy in mitigating motor fluctuations (Kaye et al., 2025; Fernandez et al., 2026). Compared with established dopamine agonists, tavapadon may provide a distinct pharmacological profile because of its preferential D1/D5 receptor activity. Conventional agents such as pramipexole mainly stimulate D2/D3 receptors, whereas rotigotine has broader activity across D1–D5 receptor subtypes. Although these agents improve motor symptoms and reduce reliance on levodopa, their clinical use is limited by adverse effects, including nausea, somnolence, orthostatic hypotension, hallucinations, peripheral edema, and impulse control disorders. The latter are particularly linked to mesolimbic dopaminergic stimulation, especially D3 receptor activation in reward-related neural circuits. By selectively engaging D1/D5 receptors, tavapadon may enhance direct-pathway striatal signaling while theoretically reducing D3-mediated behavioral adverse effects. However, this potential advantage remains inferential, and long-term head-to-head studies are needed to determine whether tavapadon has a lower risk of impulse control disorders or neuropsychiatric complications than pramipexole, ropinirole, or rotigotine. To circumvent first-pass metabolism and stabilize plasma concentrations, novel delivery platforms for dopamine agonists have advanced rapidly. Transdermal systems, including the established rotigotine patch and a ropinirole patch, provide sustained drug release and non-inferior efficacy compared to oral extended-release tablets (Raeder et al., 2021; Isaacson et al., 2023; Hattori et al., 2020a). For parenteral administration, rotigotine-loaded biodegradable polyoxazoline microspheres enable once-weekly subcutaneous dosing, with a clinical trial currently assessing its use in treatment-naïve early PD patients (Sun et al., 2024). Selective dopamine antagonists are emerging for levodopa-induced dyskinesia. Mesdopetam, a novel D3 receptor antagonist, is under clinical evaluation for dyskinesia management. Preclinical data indicate it attenuates dyskinetic movements without exacerbating underlying parkinsonian motor deficits, positioning it as a promising targeted adjunctive therapy (Olanow et al., 2020).

### 2.3 Novel MAO-B inhibitors and COMT inhibitors

Recent advances in enzyme inhibition therapy focus on multi-target modulation and optimized pharmacokinetics to enhance symptomatic control while minimizing adverse effects. Safinamide, a reversible monoamine oxidase B (MAO-B) inhibitor with additional sodium and calcium channel-blocking properties, attenuates glutamate release and serves as an effective adjunct to levodopa for reducing motor fluctuations (Shim et al., 2026; Nishikawa et al., 2025; Hattori et al., 2020b). Unlike first- (selegiline) and second-generation (rasagiline) MAO-B inhibitors—whose monotherapy efficacy in early PD exhibits considerable interindividual variability—safinamide’s dual mechanism offers complementary benefits in advanced disease (Chang et al., 2022). Combination strategies aim to leverage synergistic mechanisms at reduced individual doses. P2B001, a fixed-dose formulation of pramipexole and rasagiline, is under development as a potential first-line option for early PD. Preliminary data indicate improved motor outcomes and quality of life with a favorable tolerability profile, supporting progression to a pivotal Phase III trial (Olanow et al., 2024). Similarly, zonisamide—an antiepileptic agent with MAO-B inhibitory, dopaminergic modulatory, and calcium channel-blocking activities—has demonstrated efficacy in reducing “off” time as levodopa adjunctive therapy and is approved for this indication in several regions (Kim et al., 2026; Goel et al., 2021; Arias-Carrión and Ortega-Robles, 2025; Essam et al., 2024). In the catechol-O-methyltransferase (COMT) inhibitor class, opicapone represents a pharmacokinetic advancement over entacapone, featuring a prolonged half-life that permits once-daily dosing and more stable levodopa exposure (Song et al., 2021; Chaudhuri et al., 2022; Ferreira et al., 2019). A key ongoing question is whether early initiation of opicapone alongside levodopa can delay the emergence of motor complications; this hypothesis is being tested in a dedicated Phase III prevention trial (Ferreira et al., 2025; Ferreira et al., 2026).

## 3 Non-dopaminergic symptom treatment drugs for Parkinson’s disease

### 3.1 Glutamate neurotransmitter system and non-dopaminergic symptom treatment drugs

Targeting non-dopaminergic neurotransmitter systems offers complementary strategies for managing levodopa-induced complications and neuropsychiatric manifestations in PD. Within the glutamatergic system, the non-selective NMDA receptor antagonist amantadine remains a cornerstone for ameliorating dyskinesia and motor fluctuations (Hauser et al., 2022; Rujirussawarawong et al., 2025). Next-generation NMDA modulation is represented by AV-101 (L-4-chlorokynurenine), a prodrug converted *in vivo* to the glycine-site antagonist 7-chlorokynurenic acid; its therapeutic potential for PD-related dyskinesia is under evaluation in a clinical trial (Bourque et al., 2022). Although several metabotropic glutamate receptor (mGluR) modulators have shown preclinical promise, clinical translation has been challenging. The mGluR5 negative allosteric modulator dipraglurant (ADX48621) demonstrated favorable safety and signal of efficacy for dyskinesia in clinical studies, prompting an ongoing clinical trial to confirm clinical benefit (Negida et al., 2021; Rascol et al., 2022; McFarthing et al., 2022). Serotonergic pathways are increasingly leveraged to address both motor and non-motor complications. The 5-HT1A receptor agonist eltoprazine has shown partial efficacy against dyskinesia in clinical trials, with a pivotal clinical study currently underway (Pinna et al., 2023; LeWitt et al., 2024). For PD-associated psychosis—a frequent complication in mid-to-late disease stages where conventional antipsychotics often worsen motor function (Stamenović et al., 2024; Rose et al., 2024; Abler et al., 2022)—pimavanserin, a selective 5-HT2A receptor inverse agonist, has demonstrated robust efficacy in improving psychotic symptoms without exacerbating parkinsonism (Akbar and Friedman, 2022). Its potential utility for other neuropsychiatric features, such as impulse control disorders, remains under investigation. Additional serotonergic candidates—including 5-hydroxytryptophan, tropisetron/ondansetron, sarizotan, and the selective 5-HT1A agonist NLX-112—are in various stages of preclinical or early clinical development, primarily targeting motor complications and dyskinesia (Brüge et al., 2025; Svenningsson et al., 2025).

### 3.2 Other neurotransmitter systems

Beyond dopaminergic, glutamatergic, and serotonergic pathways, several additional neurotransmitter systems represent promising targets for symptomatic management in PD. Adenosine A2A receptor antagonism theoretically suppresses indirect pathway activity in the basal ganglia, thereby mitigating motor fluctuations (Mori et al., 2022; Isaacson and Jenner, 2025). To date, istradefylline remains the sole A2A antagonist approved for clinical use (Hauser et al., 2021); other candidates in this class have shown limited efficacy or were discontinued due to safety concerns. Cholinergic modulation has been explored primarily for gait and postural instability. Small clinical studies and case reports suggest that acetylcholinesterase inhibitors (donepezil, rivastigmine) may improve freezing of gait in PD (Chen et al., 2021). A randomized controlled trial supports rivastigmine’s benefit on postural balance (Neumann et al., 2021), and its effects on motor function, cognition, and fall prevention is under evaluating (Neumann et al., 2021). In contrast, nicotinic and muscarinic receptor-targeted agents have yielded mixed results: the α4β2 nicotinic agonist improved levodopa efficacy and reduced dyskinesia in preclinical models but failed to replicate these benefits in a clinical trial (Di Paolo et al., 2014; Trenkwalder et al., 2016). Conversely, the muscarinic M1 positive allosteric modulator TAK-071 enhanced attention and complex motor control in animal studies, prompting an ongoing Phase II trial in PD patients with gait impairment (Shanbhag et al., 2025). Noradrenergic strategies leverage the link between attentional deficits and freezing of gait. Norepinephrine reuptake inhibitors (atomoxetine, methylphenidate), established in attention-deficit/hyperactivity disorder, have shown preliminary signals of gait improvement in small observational PD studies (Wu et al., 2021). However, validation in large randomized controlled trials is still required. Finally, the GABAA receptor positive allosteric modulator zuranolone, primarily developed for depression, demonstrated exploratory efficacy against PD-related tremor in early-phase studies (Bullock et al., 2021), warranting further investigation in rigorously designed trials.

## 4 Targeted disease-modifying drugs for Parkinson’s disease

### 4.1 α-Synuclein–targeted agents

α-Synuclein (α-syn) regulates vesicular trafficking and synaptic plasticity but becomes pathogenic upon misfolding into neurotoxic oligomers and fibrils (Prahl and Coetzee, 2022; Calabresi et al., 2023; Gao et al., 2025; Geibl et al., 2024). Its prion-like intercellular propagation drives the progressive spread of pathology across neural networks, positioning extracellular α-syn clearance as a rational disease-modifying strategy (Wu and Schekman, 2024; Rahayel et al., 2022). Passive immunotherapy has advanced through several clinical stages. Two monoclonal antibodies, PRX002 and BIIB054, demonstrated favorable safety, tolerability, and cerebrospinal fluid penetration in Phase I trials (Jankovic et al., 2018; Brys et al., 2019). PRX002 subsequently progressed to clinical evaluation, which remain ongoing to assess long-term disease modification (Nikolcheva et al., 2025). In contrast, BIIB054 was terminated after failing to meet its primary motor efficacy endpoint (Lang et al., 2022). Parallel efforts in active immunization utilize synthetic peptide vaccines such as Affitope PD01 and PD03, which mimic conformational α-syn epitopes to elicit targeted humoral responses (Alfaidi et al., 2024). Early clinical data indicate acceptable safety and immunogenicity, with clinical trials under development (Volc et al., 2020). To accelerate translational harmonization, the consortium has been established to coordinate vaccine design, biomarker validation, and endpoint standardization for α-syn-targeted immunotherapies (Nimmo et al., 2021).

Targeting α-synuclein conformational stability offers a complementary disease-modifying approach. The c-Abl tyrosine kinase pathway, which negatively regulates Parkin-mediated proteostasis, has been implicated in promoting α-syn misfolding, positioning kinase inhibition as a rational therapeutic strategy (Motaln et al., 2023). Nilotinib, a second-generation tyrosine kinase inhibitor approved for chronic myeloid leukemia, demonstrates blood–brain barrier penetration and was initially evaluated for PD repurposing (Liu Y. et al., 2025; Jabbour and Kantarjian, 2024). An exploratory study reported favorable tolerability, elevated CSF dopamine metabolites, reduced α-syn oligomer levels, and improved motor scores (Pagan et al., 2016; Pagan et al., 2021). However, these promising signals were not replicated in a subsequent randomized controlled trial, which failed to confirm clinical or biomarker efficacy (Simuni et al., 2021), underscoring the translational limitations of non-selective kinase inhibition. In contrast, UCB0599 is a brain-penetrant oral small molecule specifically engineered to stabilize α-synuclein conformation and prevent pathological aggregation. Preclinical studies demonstrate that chronic administration reduces α-syn accumulation, restores mitochondrial dopamine transporter function, and ameliorates motor deficits in transgenic models (Price et al., 2018). Phase I trials established acceptable safety and pharmacokinetic profiles in both healthy volunteers and PD patients (Smit et al., 2022). A clinical trial is currently underway to evaluate its disease-modifying potential through standardized cognitive endpoints and dopaminergic terminal integrity via dopamine transporter imaging (Smit et al., 2022).

### 4.2 Glucocerebrosidase-targeted therapeutics

Mutations in the glucocerebrosidase gene (GBA1), encoding a lysosomal enzyme that hydrolyzes glucosylceramide to ceramide and glucose, represent the most prevalent genetic risk factor for parkinson’s disease (Baden et al., 2023; Ging et al., 2024; Pachchek et al., 2023). GBA1 deficiency impairs lysosomal-autophagic flux, promotes α-synuclein aggregation, and accelerates neurodegeneration, with mutation carriers typically exhibiting faster motor progression and earlier cognitive decline (Zheng and Fan, 2022; Zhang et al., 2024), (Tonin et al., 2025). Enhancing GBA enzymatic activity or reducing glucosylceramide accumulation thus constitutes a rational disease-modifying strategy. Ambroxol, a mucolytic agent repurposed as a GBA molecular chaperone, enhances enzyme trafficking and lysosomal function in GBA1-mutant cellular models (Mullin et al., 2020; Cyske et al., 2024). In the open-label AiM-PD study, ambroxol improved motor scores irrespective of GBA1 status, supporting further evaluation (Mullin et al., 2020). Its effects on cognitive and motor trajectories in PD is under a clinical trial (Toffoli et al., 2026). Similarly, LTI-291, a selective GBA activity modulator, demonstrated favorable tolerability and increased GBA activity in plasma and CSF among GBA1-mutation carriers during clinical testing; however, clinical efficacy endpoints remain to be established (den Heijer et al., 2023). For patients with severe enzymatic deficiency, gene replacement offers a direct corrective approach. PR001A, an AAV-based vector delivering functional GBA1, requires intracerebral administration and is under Phase I/IIa evaluation for safety and target engagement in GBA1-associated PD (Abeliovich et al., 2021). Conversely, substrate reduction therapy aims to limit glucosylceramide accumulation upstream. The glucosylceramide synthase (GCS) inhibitor venglustat reduced peripheral glucosylceramide levels and showed acceptable safety in a Phase II trial but failed to meet its primary endpoint of motor improvement (Giladi et al., 2023), highlighting the challenge of translating biochemical target engagement into clinical benefit.

### 4.3 Neuroprotective drugs

#### 4.3.1 Glucagon-like Peptide-1 and Sigma-1 receptor agonists

Impaired insulin signaling cascades in the central nervous system contribute to neuronal metabolic stress and neurodegeneration, providing a rationale for repurposing antidiabetic glucagon-like peptide-1 (GLP-1) receptor agonists as neuroprotective agents in Parkinson’s disease (Li et al., 2026). These compounds exert pleiotropic effects beyond glycemic control, including mitochondrial bioenergetic enhancement, autophagy induction, microglial phenotype modulation, and facilitation of pathogenic protein clearance (Gandhi and Parhizgar, 2025; Hong et al., 2024; Urkon et al., 2025), (Hong et al., 2024; Urkon et al., 2025; Athauda et al., 2017). The most compelling clinical evidence derives from exenatide. A clinical randomized trial in PD demonstrated not only motor symptom improvement but also DAT-SPECT evidence suggesting attenuated nigrostriatal dopaminergic decline, supporting potential disease-modifying activity (Athauda et al., 2017). These findings have prompted a pivotal, double-blind clinical trial designed to definitively assess exenatide’s impact on clinical progression (Vijiaratnam et al., 2021). The therapeutic pipeline continues to expand with next-generation GLP-1 receptor agonists featuring diverse pharmacokinetic profiles. Semaglutide, liraglutide, and lixisenatide—each with distinct blood–brain barrier permeability and receptor binding kinetics—are under Phase II evaluation in PD (Lv et al., 2024). Additionally, novel formulations such as the sustained-release exenatide depot (PT320) and the brain-penetrant candidate LNY01 aim to optimize central target engagement while minimizing peripheral adverse effects. Collectively, these efforts seek to validate GLP-1 receptor activation as a viable disease-modifying strategy and to define optimal dosing regimens for neuroprotection in PD.

The sigma-1 receptor (Sig-1R) operates as a ligand-regulated molecular chaperone primarily enriched at mitochondria-associated endoplasmic reticulum membranes (MAMs) (Shi et al., 2021; Ngo et al., 2024). Through this strategic localization, Sig-1R fine-tunes intracellular calcium homeostasis, attenuates oxidative stress, and amplifies neurotrophic signaling cascades (Ahmed et al., 2025; Shi et al., 2025). Pharmacological activation further reinforces cellular proteostatic networks by stimulating autophagy and proteasomal clearance, offering a mechanistic rationale for mitigating α-synuclein accumulation and dopaminergic neuronal loss in Parkinson’s disease (Yin et al., 2025). Clinical validation of this target has advanced with blarcamesine, a selective Sig-1R agonist. In a clinical trial evaluating patients with Parkinson’s disease dementia, blarcamesine demonstrated a favorable safety profile and successfully met both primary and secondary efficacy endpoints (Reyes et al., 2021). These encouraging cognitive and functional outcomes have accelerated further clinical development to delineate its broader disease-modifying utility across PD phenotypes (Table 1).

**TABLE 1:** Emerging pharmacological strategies for Parkinson’s disease.

Pharmacological strategies	Representative agents	Primary targets	Clinical stage	Advantage
Optimized dopaminergic formulations	CVT-301, IPX066, ND0612, AP-CD/LD	Rapid or continuous levodopa delivery; stabilization of plasma dopamine	Approved/Phase III	Reduced “off” time; rapid rescue
Novel dopamine receptor modulators	Tavapadon, mesdopetam	Selective D1/D5 agonism; D3 antagonism	Phase II–III	Improved motor control with fewer impulse effects
MAO-B/COMT inhibitors	Safinamide, opicapone, P2B001	Dopamine metabolism inhibition; glutamate modulation	Approved/Phase III	Once-daily dosing; adjunct benefit
Glutamatergic modulators	Amantadine, AV-101, dipraglurant	NMDA or mGluR5 inhibition	Phase II–III	Dyskinesia control
Serotonergic agents	Pimavanserin, eltoprazine	5-HT2A inverse agonism; 5-HT1A agonism	Approved/Phase II–III	Improves psychosis without motor worsening
Adenosine A2A antagonists	Istradefylline	Basal ganglia indirect pathway inhibition	Approved	Reduces “off” episodes
α-Synuclein immunotherapy	PRX002, BIIB054, PD01/PD03	Clearance of extracellular α-synuclein	Phase I–II	Strong mechanistic rationale
α-Synuclein aggregation inhibitors	Nilotinib, UCB0599	c-Abl inhibition; misfolding suppression	Phase I–II	BBB penetration
GBA-targeted therapies	Ambroxol, LTI-291, PR001A, venglustat	Lysosomal enhancement; lipid metabolism	Phase I–II	Precision medicine approach
Neuroprotective agents	Exenatide, blarcamesine, deferiprone, UDCA	Mitochondrial, anti-inflammatory, metabolic modulation	Phase II–III	Disease-modifying potential

#### 4.3.2 Antioxidative stress–targeted drugs

**FIGURE 1:**
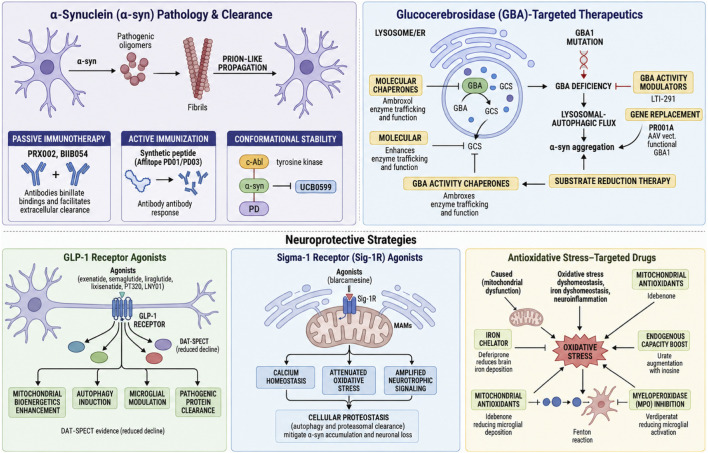
Current and emerging pharmacotherapies in Parkinson’s disease.

Oxidative stress, driven by mitochondrial dysfunction, iron dyshomeostasis, and neuroinflammation, represents a central pathogenic mechanism in Parkinson’s disease and a rational target for disease modification (López-Aguirre et al., 2025). Iron accumulation in the substantia nigra exacerbates Fenton reaction–mediated oxidative damage. The iron chelator deferiprone reduced brain iron deposition and improved motor scores in an open-label clinical study (Martin-Bastida et al., 2017). However, the subsequent randomized clinical trial, while confirming target engagement (reduced brain iron), reported worsened clinical outcomes, highlighting the complexity of iron modulation in neurodegeneration (Devos et al., 2022). Mitochondrial antioxidants have also been pursued. Idebenone, a synthetic coenzyme Q10 analogue with enhanced bioavailability, demonstrates neuroprotective effects in PD models (Yan et al., 2022; Zhang et al., 2026). Three parallel Phase II trials in China are currently evaluating idebenone’s impact on disease progression in early PD, α-synuclein–associated REM sleep behavior disorder, and combined motor/non-motor symptoms (Li et al., 2021; Li et al., 2022). Elevating endogenous antioxidant capacity via urate augmentation represents another strategy. Epidemiological data link higher serum urate levels to reduced PD risk, though hyperuricemia carries gout and nephrolithiasis risks (Liu and Reynolds, 2025; Liu X. et al., 2025). The urate precursor inosine successfully raised serum urate and showed biomarker signals of neuroprotection in the SURE-PD study (Parkinson Study Group SURE-PD3 Investigators et al., 2021). Nevertheless, the pivotal clinical trial failed to demonstrate clinical benefit in early PD, underscoring the challenge of translating surrogate biomarker effects into meaningful disease modification (Parkinson Study Group SURE-PD3 Investigators et al., 2021). Finally, inhibition of myeloperoxidase (MPO)—an enzyme that amplifies reactive oxygen species generation and is upregulated in PD-affected brain regions—offers an anti-inflammatory antioxidative approach. The MPO inhibitor verdiperstat reduced microglial activation on 11C-PBR28 PET imaging in a clinical study (Jucaite et al., 2015; Espejo et al., 2025) (Figure 1).

## 5 Conclusion

Pharmacological intervention remains the cornerstone of Parkinson’s disease (PD) management and continues to represent the most widely adopted therapeutic strategy in clinical practice. At present, PD treatment is largely centered on dopamine replacement–based symptomatic therapy. Several novel dopaminergic formulations and non-dopaminergic agents have been approved or are under clinical development, substantially expanding the therapeutic landscape. Nevertheless, disease-modifying therapy remains the most critical unmet need in PD, as the majority of candidate agents are still confined to early-phase clinical trials.

Notably, a considerable proportion of previous clinical studies have been limited by suboptimal trial design, including insufficient alignment between drug mechanisms and therapeutic targets, high heterogeneity of enrolled populations, inappropriate or insensitive outcome measures, and the lack of objective and quantitative tools to assess disease progression. These limitations have, in part, hindered the successful translation of promising disease-modifying strategies into clinical benefit. Future trials should prioritize methodological refinement, with greater emphasis on patient stratification, rational inclusion criteria, and mechanism-driven endpoint selection. In particular, stratifying patients based on clinical phenotypes, genetic background, or disease stage may enhance the precision and interpretability of therapeutic evaluation.
